# De-Regulation of JNK and JAK/STAT Signaling in *ESCRT-II* Mutant Tissues Cooperatively Contributes to Neoplastic Tumorigenesis

**DOI:** 10.1371/journal.pone.0056021

**Published:** 2013-02-13

**Authors:** Sarah E. Woodfield, Hillary K. Graves, Jacob A. Hernandez, Andreas Bergmann

**Affiliations:** 1 Graduate Program in Developmental Biology, Baylor College of Medicine, Houston, Texas, United States of America; 2 Department of Biochemistry and Molecular Biology, The University of Texas M.D. Anderson Cancer Center, Houston, Texas, United States of America; 3 Department of Cancer Biology, University of Massachusetts Medical School, Worcester, Massachusetts, United States of America; National Cancer Institute, United States of America

## Abstract

Multiple genes involved in endocytosis and endosomal protein trafficking in *Drosophila* have been shown to function as neoplastic tumor suppressor genes (nTSGs), including Endosomal Sorting Complex Required for Transport-II (ESCRT-II) components *vacuolar protein sorting 22* (*vps22*), *vps25*, and *vps36*. However, most studies of endocytic nTSGs have been done in mosaic tissues containing both mutant and non-mutant populations of cells, and interactions among mutant and non-mutant cells greatly influence the final phenotype. Thus, the true autonomous phenotype of tissues mutant for endocytic nTSGs remains unclear. Here, we show that tissues predominantly mutant for *ESCRT-II* components display characteristics of neoplastic transformation and then undergo apoptosis. These neoplastic tissues show upregulation of c-Jun N-terminal Kinase (JNK), Notch, and Janus Kinase (JAK)/Signal Transducer and Activator of Transcription (STAT) signaling. Significantly, while inhibition of JNK signaling in mutant tissues partially inhibits proliferation, inhibition of JAK/STAT signaling rescues other aspects of the neoplastic phenotype. This is the first rigorous study of tissues predominantly mutant for endocytic nTSGs and provides clear evidence for cooperation among de-regulated signaling pathways leading to tumorigenesis.

## Introduction

Tumor development involves destabilization of the well-controlled processes of cell proliferation, cell polarization, and programmed cell death that are tightly regulated by widely conserved signaling pathways. Therefore, genes that act as regulators of these signaling pathways may behave as nTSGs. In *Drosophila*, as well as in other organisms, genes that control endocytosis and endosomal protein sorting behave as nTSGs. Such endocytic nTSGs include *avalanche* (*avl*) [1], *Rab5* [1], *vps45* [2], *Rabenosyn* (*Rbsn*) [2], *tumor suppressor protein 101* (*tsg101* aka *erupted* (*ept*) or *vps23*) [3], *vps28* [4], *vps25* [5]–[7], *vps22* (aka *larsen* (*lsn*)) [8], *vps20* [4], *shrub* (*shrb*) [4], *vps2* [4], and *vps4* [9]. These endocytic nTSGs are involved in endocytosis and endosomal protein sorting of cell signaling receptors and other membrane proteins and inhibit tumor formation by ensuring proper trafficking and collection of cargoes that function in growth control, cell survival, and apical-basal polarity in epithelial tissues.

The ESCRT machinery promotes the maturation of early endosomes into multi-vesicular bodies (MVBs) [10]–[12]. This is a complex process that involves four ESCRT complexes, ESCRT-0, -I, -II and -III. Of interest to this study are the ESCRT-II components *vps22*, *vps25,* and *vps36*. The products of these genes mediate the transfer of cargo from ESCRT-I to ESCRT-III [10]–[12]. Loss-of-function mutations of these genes block this process, which causes abnormal signaling and triggers a complex phenotype composed of autonomous and non-cell autonomous effects [5]–[8].

Previous studies of the mutant phenotypes of *ESCRT-II* components and other endocytic nTSGs focused on their mosaic phenotype, when mutant clones are surrounded by wild-type cells. Thus, the complex mosaic phenotype of endocytic nTSGs has been well characterized. Epithelial polarity and proliferation control are disrupted in mutant clones [1], [3], [7], [9]. Mutant clones in eye-antennal imaginal discs fail to express the neuronal marker ELAV, indicating that they fail to differentiate [6], [7]. A clear non-cell autonomous effect of mutant clones on proliferation is observed in tissues mosaic for *tsg101*, *vps22*, or *vps25* [3], [5], [7], [8]. The non-mutant tissues surrounding the mutant clones display increased proliferation [3], [5], [7]. Such tissues form multilayered discs and overgrown adult structures [1], [3], [5]–[7]. *vps25* mutant clones also promote non-cell autonomous cell survival through upregulation of the apoptosis inhibitor Diap1 [5], [8], [13].

In mutant clones of endocytic nTSGs, endosomal trafficking is blocked and membrane proteins accumulate in abnormal endosomal compartments [1], [3], [5]–[9], [14]. For example, Notch protein accumulates in abnormally enlarged early endosomes where it undergoes ligand-independent processing and activation [1]–[8]. Active Notch induces non-cell autonomous proliferation in *vps22, vps25,* and *tsg101* mosaic tissues through non-cell autonomous upregulation of JAK/STAT and Yorkie signaling [3], [5], [7], [8], [13].

In mosaic tissues, mutant clones of *tsg101* and *vps25* are apoptotic [3], [5]–[7]. Apoptosis in these clones is induced by JNK signaling and the canonical apoptotic pathway (Hid/Diap1/Dronc/Ark) [5], [9], [15]. It is commonly believed that JNK signaling and thus apoptosis is induced by cell competition from neighboring non-mutant tissue [6], [16]. Inhibition of apoptosis in *vps25* mutant clones unleashes a strong neoplastic phenotype characterized by massive tumorous overgrowth, loss of cell polarity, and invasive properties [5], [6]. Thus, apoptosis serves as a tumor suppressor mechanism. A strong neoplastic phenotype is also observed when the entire tissue is mutant for nTSGs, thus when competitive interactions between mutant and non-mutant tissues are eliminated [8], [17].

From these studies, it is clear that the interactions between the mutant and non-mutant populations of cells greatly influence the final phenotype. However, while the non-cell autonomous mechanisms that cause hyperplastic overgrowth are well characterized, the mechanisms that cause autonomous neoplastic transformation of tissue mutant for endocytic nTSGs are poorly understood. Because endocytic trafficking controls multiple signaling pathways, it is likely that tumors caused by mutations in endocytic nTSGs acquire their neoplastic characteristics through the de-regulation of numerous signaling pathways. In hypomorphic *tsg101* and *vps25* mutant clones, Yorkie signaling is up-regulated [5], [13], [15]. However, in strong (null) *vps25* mosaic discs, Yorkie signaling is only detectable non-cell autonomously in non-mutant neighboring cells [13], suggesting that Yorkie signaling does not significantly contribute to the neoplastic phenotype of these mutant clones.

In endocytic nTSG mutant tissues, the protein levels of the JAK/STAT ligand Unpaired (Upd), the JAK/STAT receptor Domeless (Dome), and the *Drosophila* STAT, Stat92E, are increased, leading to increased JAK/STAT signaling activity [3], [6], [7], [18]. However, the role of JAK/STAT signaling for the autonomous neoplastic phenotype of nTSG mutant tissue is less clear. Early evidence has indicated that JAK/STAT signaling may be involved in this neoplastic transformation; however, that experiment was done in a heterozygous *Stat92E* condition throughout the disc that affects both autonomous and non-cell autonomous phenotypes [18]. A rigorous assessment of the neoplastic phenotype in predominantly nTSG mutant tissue in which JAK/STAT signaling is disrupted has not been performed yet.

Here, in order to understand the cause of the neoplastic transformation of these mutant clones, we employed the *ey-FLP/cell lethal* (*cl*) system [19] to generate predominantly mutant tissues of the ESCRT-II components *vps22*, *vps25* and *vps36*. These overgrown, neoplastic tumors display disorganized cellular architecture and disrupted epithelial structures with expanded apical-basal domains. Additionally, these tissues are unable to terminally differentiate and are invasive. Unexpectedly, although competitive cellular interactions have been largely eliminated by the *ey*-*FLP*/*cl* method, these predominantly mutant tissues are also very apoptotic. Within mutant tissues, JNK, Notch, and JAK/STAT signaling are up-regulated. Reducing JNK activity in *ESCRT-II* mutant tissue partially blocks the overproliferation phenotype and apoptosis but does not otherwise affect neoplastic transformation. In addition, complete loss of JAK/STAT signaling strongly rescues the neoplastic phenotype. Thus, this study supports the idea that de-regulation of signaling pathways, especially JNK and JAK/STAT signaling, in *vps22*, *vps25*, and *vps36* mutant tissues leads to neoplasia.

## Materials and Methods

### *Drosophila* Genetics and Generation of Predominantly Mutant Imaginal Discs

The following mutants and transgenic lines were used: *vps22^5F3-8^* [20], *vps25^N55^* [5], *vps36^Δ69^*(this study), *ark^H16^* [21], *Stat92E^397^* [22], *puc-lacZ* [23], *Gbe-Su(H)-lacZ* [24], *E(spl)m8 2.61-lacZ* [25], *10X-STAT-GFP* [26], *UAS-bsk^DN^* [27], and *ey-Gal4* [28]. *vps36^Δ69^* is a null allele generated by imprecise excision of the P-element transposon inserted in the first exon 29 base pairs upstream of the initiator ATG in the *vps36^L5212^* allele.

To generate imaginal discs predominantly mutant for *vps22*, *vps25*, or *vps36*, we used the *ey-FLP/cl* technique [19], [28]. *cl* indicates an anonymous cell lethal mutation that kills cells when homozygous [19], [28]. The *ESCRT-II* mutant alleles were crossed to *ey-FLP; FRT cl* flies. The use of the *FRT* depended on the location of the *ESCRT-II* gene in the genome. The complete genotypes are indicated in the legends to the figures.

### Immunohistochemistry

Imaginal discs were dissected from third instar larvae and stained using standard protocols. The following antibodies were used: mouse α-Dlg (1∶20), rat α-ELAV (1∶40), mouse α-Mmp1 (catalytic domain; 1∶50), and mouse α-Notch^intra^ (1∶20; DSHB, University of Iowa); mouse α-BrdU (1∶50; Becton Dickinson); rabbit α-cleaved Caspase-3 (Cas-3*; 1∶500; Cell Signaling Technology); mouse α-β-gal (1∶1,000) and rabbit α-pJNK (1∶100; Promega); and rabbit α-aPKC (1∶100; Santa Cruz Biotechnology). AF488-phalloidin and AF546-phalloidin were obtained from Sigma Aldrich. Cy-3 and Cy-5 fluorescently-conjugated secondary antibodies were obtained from Jackson ImmunoResearch. Vectashield with DAPI was obtained from Vector Laboratories. TUNEL kit was obtained from Roche Diagnostics. Images were captured using Olympus Optical FV500 or FV1000 confocal microscopes and processed using Adobe Photoshop CS4.

## Results

### *ESCRT-II* Mutant Tissues Show Neoplastic Characteristics

The *ey-FLP/cl* method generates eye-antennal imaginal discs that are almost entirely composed of mutant tissue in otherwise heterozygous animals (see Methods) [19], [28]. This is accomplished by elimination of the twin-spots after *ey-FLP-*induced mitotic recombination by a cell lethal (cl) mutation that is present on the homologous chromosome arm. The use of the *ey-FLP* ensures high FLP activity such that most cells undergo mitotic recombination and only a few heterozygous cells remain. Thus, eye-antennal discs generated by this method are almost entirely mutant for the gene of interest.

**Figure 1:**
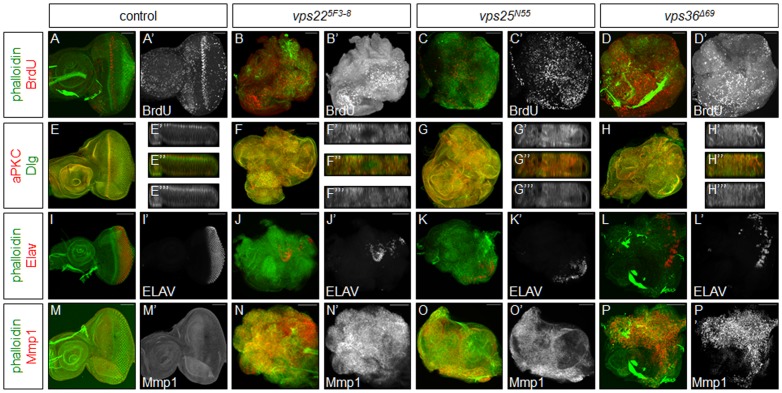
Tissues predominantly mutant for ESCRT-II components *vps22*, *vps25*, or *vps36* show neoplastic characteristics. Shown are predominantly mutant eye-antennal imaginal discs. Phalloidin (green) is used to mark the overall shape of the tissue. Scale bars represent 50 µm. (**A–D**) BrdU (red and grayscale) labelings show that proliferation is increased in discs predominantly mutant for *vps22* (B,B’), *vps25* (C,C’), or *vps36* (D,D’), as compared to proliferation in control discs (A,A’). (**E–H**) aPKC (red and grayscale (E’,F’,G’,H’)) and Dlg (green and grayscale (E’’’,F’’’,G’’’,H’’’)) labelings of discs predominantly mutant for *vps22* (F–F’’’), *vps25* (G–G’’’), or *vps36* (H–H’’’) show that cellular architecture is disrupted, as compared to the architecture of control discs (E–E’’’). (**I–L**) ELAV (red and grayscale) labelings of discs predominantly mutant for *vps22* (J,J’), *vps25* (K,K’), or *vps36* (L,L’) show that very few cells in the mutant discs differentiate normally, as compared to differentiation in control discs (I,I’). (**M–P**) Mmp1 (red and grayscale) labelings of discs predominantly mutant for *vps22* (N,N’), *vps25* (O,O’), or *vps36* (P,P’) show that levels of this protein are elevated, as compared to Mmp1 levels in control discs (M,M’). **Genotypes**: (**A**) *eyFLP;; FRT82B/FRT82B cl*. (**E,I,M**) *eyFLP; FRT42D y^+^/FRT42D cl*. (**B,F,J,N**) *eyFLP;; FRT82B vps22^5F3-8^/FRT82B cl*. (**C,G,K,O**) *eyFLP; FRT42D vps25^N55^ y^+^/FRT42D cl*. (**D,H,L,P**) *eyFLP;; vps36^Δ69^ FRT80B/cl FRT80B*.

We used the *ey-FLP/cl* system to generate tissues predominantly mutant for ESCRT-II components *vps22*, *vps25*, or *vps36*. These predominantly mutant epithelial tissues have a very striking phenotype: unlike wild-type single-layered eye-antennal imaginal discs, they overgrow into multi-layered, dense “balls” of cells (Figure 1) [7], [8], [17]. These discs also vary considerably in size. Some are about the size of wild-type discs or even slightly smaller while others can be three to five times as large [8], [17]. This was also reported for other endocytic nTSGs [2], [17]. To understand this tumor-like phenotype in more detail, we examined proliferation, cellular architecture, differentiation, and metastatic potential of eye-antennal discs predominantly mutant for *vps22*, *vps25*, or *vps36*.

To assay proliferation in the predominantly mutant tissues, we used Bromodeoxyuridine (BrdU) labeling to mark cells in S-phase. Control discs show the normal BrdU pattern in eye-antennal discs (Figure 1A). Of note is the posterior part of the eye disc in which cells are post-mitotic and differentiate into photoreceptor neurons, cone cells, and other cell types. In discs predominantly mutant for *ESCRT-II* components, BrdU labeling indicates that proliferation is occurring at elevated levels throughout the entire disc (Figure 1B–D). Post-mitotic areas are not visible or are very small. Thus, proliferation is up-regulated in tissues predominantly mutant for *vps22*, *vps25*, or *vps36*.

To examine cellular architecture of tissues predominantly mutant for *ESCRT-II* components, we first labeled discs with phalloidin. Phalloidin recognizes cortical actin and thus highlights cellular architecture and organization throughout tissues. Control discs stained with phalloidin show a consistent shape characteristic of *Drosophila* eye-antennal imaginal discs (Figure 1A,I,M). Discs predominantly mutant for *ESCRT-II* components trade this characteristic shape for a disorganized, expanded, amorphic structure in which the antennal and eye portions of the disc cannot be distinguished (Figure 1B–D,J–L,N–P). Next, to specifically examine epithelial polarity, we labeled predominantly mutant tissues with antibodies recognizing atypical Protein Kinase C (aPKC) and Discs large (Dlg). In control tissue, aPKC localizes to the polarized apical membrane domain while Dlg is found at the basolateral membrane domain (Figure 1E). In the predominantly mutant tissues, aPKC and Dlg are spread outside of their respective regions of wild-type localization (Figure 1F–H), indicating that apical-basal polarity is disrupted. Together, these data indicate that cellular architecture is disrupted in *vps22*, *vps25*, and *vps36* mutant tissues, which is consistent with previous reports [7], [8], [17].

It has been shown previously that clones of *vps25* mutant cells in mosaics fail to differentiate [6], [7]. Therefore, we were curious to examine the ability of cells to differentiate if almost the entire eye-antennal disc is mutant. Photoreceptor neurons are the first cells that differentiate during eye development. Using ELAV as a neuronal marker, we labeled eye-antennal discs almost entirely mutant for *ESCRT-II* components to assess differentiation. In the control eye-antennal imaginal disc, cells in the posterior of the disc differentiate into neurons and thus show high expression of ELAV (Figure 1I). In contrast, very few cells in the *ESCRT-II* predominantly mutant tissues show ELAV expression (Figure 1J–L). The cells that are positive for ELAV are not localized to a specific region of the disc but rather are scattered throughout the tissue. Thus, similar to mutant cells in a mosaic background, cells in predominantly mutant eye-antennal imaginal discs fail to differentiate. The few cells that do differentiate likely correspond to the few heterozygous cells that are present in the disc.

Loss of epithelial integrity and apical-basal polarity, increased proliferation, and loss of differentiation are hallmarks of neoplastic transformation. It has also been demonstrated that *vps25* mutant cells have invasive behavior [6]. Matrix metalloprotease 1 (Mmp1) remodels the extracellular matrix and is known to be elevated in and required for metastasis of *Drosophila* tumors [29]. Therefore, to correlate the metastatic potential of the predominantly mutant *vps22*, *vps25*, and *vps36* discs with Mmp1 expression, we labeled these discs with an antibody recognizing Mmp1. In control eye-antennal imaginal discs, Mmp1 is present at very low levels (Figure 1M). In contrast, in the predominantly mutant discs, Mmp1 is present at high levels throughout the discs (Figure 1N–P). Taken together, these data demonstrate that ESCRT-II components *vps22*, *vps25*, and *vps36* are strong nTSGs and that eye-antennal imaginal discs predominantly mutant for these genes display neoplastic characteristics.

### Notch, JAK/STAT, and JNK Signaling are Up-regulated in *ESCRT-II* Mutant Tissues

Due to the endosomal sorting defect in *ESCRT-II* mutant tissues, multiple signaling pathways are de-regulated [4]–[8], [14], [15]. In discs mosaic for *ESCRT-II* mutants, it is well understood how de-regulation of signaling contributes to the non-cell autonomous proliferation and survival phenotypes (see Introduction). However, these studies in mosaic tissues fail to answer two important questions: (1) What signaling pathways are de-regulated in predominantly mutant tissues completely independent from interactions with non-mutant populations of cells? (2) Does this autonomous de-regulation of signaling contribute to the autonomous neoplastic phenotype? To answer the first question, we examined levels of Notch, JAK/STAT, and JNK signaling in discs predominantly mutant for *ESCRT-II* components.

**Figure 2:**
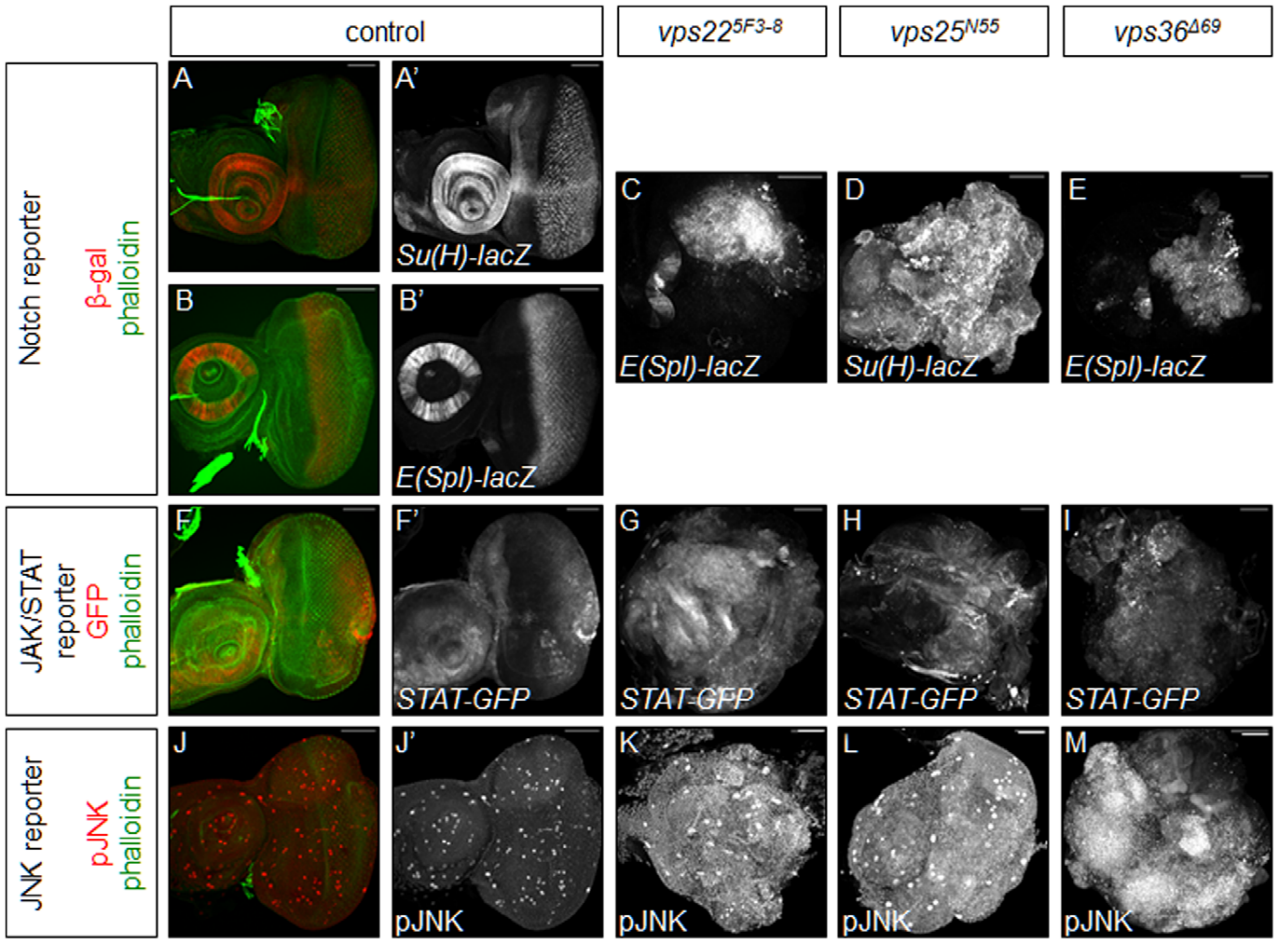
Notch, JAK/STAT, and JNK signaling are upregulated in *vps22*, *vps25*, and *vps36* mutant tissues. Shown are predominantly mutant eye-antennal imaginal discs. Phalloidin (green) is used to mark the overall shape of the tissue. *Gbe-Su(H)-lacZ*, and *E(spl)m8 2.61-lacZ* are detected by β-gal labeling (red or grayscale). Scale bars represent 50 µm. (**A,E**) Imaginal discs predominantly mutant for *vps25* induce high levels of *Gbe-Su(H)-lacZ* (E), as compared to control discs (A,A’). (**B,C,E**) Imaginal discs predominantly mutant for *vps22* (C) or *vps36* (E) induce high levels of *E(spl)m8 2.61-lacZ*, as compared to control discs (B,B’). (F–I) Imaginal discs predominantly mutant for *vps22* (G), *vps25* (H), or *vps36* (I) induce high levels of *10X-STAT-GFP*, as compared to control discs (F,F’). (J–M) Imaginal discs predominantly mutant for *vps22* (K), *vps25* (L), or *vps36* (M) show high levels of phosphorylated JNK protein, as compared to control discs (J,J’). **Genotypes**: (**A**) *eyFLP; FRT42D y^+^/FRT42D cl; Gbe-Su(H)-lacZ/+.* (**B**) *eyFLP; E(Spl)m8 2.61-lacZ/+; FRT82B/FRT82B cl.* (**C**) *eyFLP; E(spl)m8 2.61-lacZ/+; FRT82B vps22^5F3-8^/FRT82B cl.* (**D**) *eyFLP; FRT42D vps25^N55^ y^+^/FRT42D cl; Gbe-Su(H)-lacZ/+.* (**E**) *eyFLP; E(spl)m8 2.61-lacZ/+; vps36^Δ69^ FRT80B/cl FRT80B.* (**F**) *eyFLP; FRT42D y^+^/FRT42D cl; 10X-STAT-GFP/+.* (**G**) *eyFLP; 10X-STAT-GFP/+; FRT82B vps22^5F3-8^/FRT82B cl.* (**H**) *eyFLP; FRT42D vps25^N55^ y^+^/FRT42D cl; 10X-STAT-GFP/+.* (**I**) *eyFLP; 10X-STAT-GFP/+; vps36^Δ69^ FRT80B/cl FRT80B.* (**J**) *eyFLP; FRT42D y^+^/FRT42D cl.* (**K**) *eyFLP;; FRT82B vps22^5F3-8^/FRT82B cl.* (**L**) *eyFLP; FRT42D vps25^N55^ y^+^/FRT42D cl.* (**M**) *eyFLP;; vps36^Δ69^ FRT80B/cl FRT80B.*

Multiple studies have shown that Notch signaling is up-regulated in tissues mosaic for *ESCRT* components [3]–[5], [7], [8], [14]. Thus, we were interested to examine levels of the Notch signaling pathway in tissues predominantly mutant for *ESCRT-II* components. To assess Notch signaling, we used two Notch reporters, the *Gbe-Su(H)-lacZ* reporter [24] and the *E(spl)m8 2.61-lacZ* reporter [25]. In control discs, Notch signaling is high in a very stereotypical pattern in the posterior of the eye disc and in the antennal disc (Figure 2A,B). Use of the *Gbe-Su(H)-lacZ* reporter in *vps25* mutant discs showed that Notch signaling is very high throughout the entire disc (Figure 2D). We used the *E(spl)-lacZ* reporter to examine Notch activity in *vps22* and *vps36* mutant tissues and found that Notch signaling is indeed very high but only in about half of each mutant disc (Figure 2C,E). To further examine Notch signaling within mutant discs, we assayed levels of the Notch protein using an antibody that recognizes the intracellular portion of the receptor. We found that protein levels are indeed very high throughout mutant discs (Figure S1B-D), supporting the results found with the *Gbe-Su(H)-lacZ* reporter. From these data, we clearly see that Notch signaling is up-regulated in tissues predominantly mutant for *ESCRT-II* components.

In genetic mosaics, increased JAK/STAT signaling has been observed in *tsg101* and *vps25* mutant clones, and Notch-induced upregulation of the JAK/STAT ligand Upd has been shown to contribute to the non-cell autonomous increase of proliferation in neighboring non-mutant cells [3], [5], [7], [8], [18]. Thus, we were interested to see if JAK/STAT signaling is affected autonomously in predominantly *ESCRT-II* mutant tissues. To assess levels of JAK/STAT signaling, we used the well-characterized *10X-STAT-GFP* reporter [26]. In control discs, JAK/STAT signaling is only active in the posterior portion of the eye disc and in the antennal disc (Figure 2F). In contrast, JAK/STAT signaling is clearly very elevated throughout *ESCRT-II* mutant discs (Figure 2G–I).

One additional pathway that is autonomously induced in mutant clones of endocytic nTSG mosaics is JNK signaling [5], [9], [15], [16], [30]. It is assumed that JNK signaling is induced by cell competition between mutant (loser) and non-mutant (winner) cells in the mosaics. In discs predominantly mutant for *ESCRT-II* genes, the competitive interaction between mutant and non-mutant tissue is removed because most of the non-mutant tissue is eliminated and only mutant tissue remains. We were therefore surprised to see strong labeling with the pJNK antibody, which detects phosphorylated and thus activated JNK, in discs predominantly mutant for *ESCRT-II* components compared to controls (Figure 2J–M). We also observed a strong induction of *puc-lacZ*, a JNK-reporter transgene, in discs predominantly mutant for *vps25* (Figure S2B). Therefore, JNK activity is induced in *ESCRT-II* mutant discs independently of cell competition. Taken together, these data show that the Notch, JAK/STAT, and JNK signaling pathways are up-regulated in predominantly *ESCRT-II* mutant tissues and support a possible role for these conserved signaling pathways in the neoplastic phenotype observed in these tissues.

### Tissues Predominantly Mutant for *ESCRT-II* Components are Apoptotic

**Figure 3:**
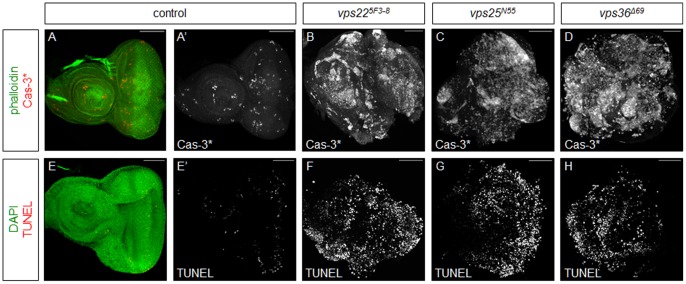
Imaginal discs predominantly mutant for *vps22*, *vps25*, or *vps36* are apoptotic. Shown are predominantly mutant eye-antennal imaginal discs. Phalloidin (green) or DAPI (green) is used to mark the overall shape of the tissue. Scale bars represent 50 µm. (**A–D**) Cleaved Caspase-3 (Cas-3*; red and grayscale) labelings show that apoptosis is increased in discs predominantly mutant for *vps22* (B), *vps25* (C), or *vps36* (D), as compared to apoptosis in control discs (A,A’). (**E–H**) TUNEL (red and grayscale) labelings show that apoptosis is increased in discs predominantly mutant for *vps22* (F), *vps25* (G), or *vps36* (H), as compared to apoptosis in control discs (E,E’). **Genotypes**: (**A**) *eyFLP; FRT42D y^+^/FRT42D cl.* (**B,F**) *eyFLP;; FRT82B vps22^5F3-8^/FRT82B cl.* (**C,G**) *eyFLP; FRT42D vps25^N55^/FRT42D cl.* (**D,H**) *eyFLP;; vps36^Δ69^ FRT80B/cl FRT80B.* (**E**) *eyFLP;; FRT82B/FRT82B cl.*

JNK signaling in *nTSG* mutant clones in mosaic discs triggers apoptosis [5], [16], [30], [31]. Thus, although competitive interactions are largely abolished in predominantly *ESCRT-II* mutant discs, which are often overgrown, we examined these discs for apoptosis. We assayed cell death by cleaved Caspase-3 (Cas-3*) and TUNEL labeling in predominantly mutant discs. In control discs, a few Cas-3* positive cells are scattered throughout the tissue, but most cells are not apoptotic (Figure 3A). However, surprisingly, discs predominantly mutant for *ESCRT-II* genes show high levels of Cas-3* throughout (Figure 3B–D). Similar results were obtained with TUNEL labeling (Figure 3E–H), which detects DNA fragmentation, a hallmark of apoptosis [32], indicating that apoptosis is indeed occurring. Taken together, although competitive interactions between mutant and non-mutant cells are eliminated in discs predominantly mutant for *ESCRT-II* components, they display high levels of apoptosis.

**Figure 4:**
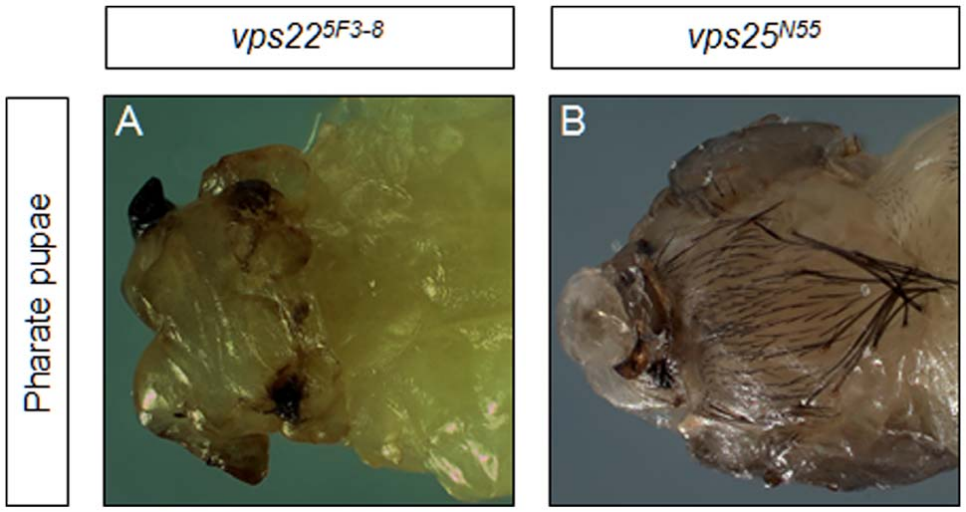
Animals with imaginal discs predominantly mutant for ESCRT-II components die as headless pharate pupae. Animals with predominantly mutant tissues are generated with the *eyFLP-cl* system. (**A,B**) Animals with eye-antennal imaginal discs predominantly mutant for *vps22* (A) or *vps25* (B) die as pharate pupae that lack heads. **Genotypes**: (**A**) *eyFLP;; FRT82B vps22^5F3-8^/FRT82B cl.* (**B**) *eyFLP; FRT42D vps25^N55^ y^+^/FRT42D cl*.

So far, we have analyzed the phenotypes of eye-antennal imaginal discs of *ESCRT-II* mutants of third instar larvae. We also observed that animals with eye-antennal imaginal discs predominantly mutant for *ESCRT-II* components die as pharate pupae. Based on our data from imaginal discs, we hypothesized that the apoptosis of the discs may contribute to the death of the pharate pupae. Dissection and examination of the pharate pupae demonstrated that they lack head structures (Figure 4A,B). Thus, it is likely that the apoptosis of the mutant tissues is leading to the death of the animal.

### Inhibition of JNK Affects the Neoplastic Transformation of *ESCRT-II* Mutant Tissues

We were curious to examine the role of apoptosis and JNK signaling in these discs. JNK is particularly interesting in this respect because under certain conditions it not only induces apoptosis, but also non-cell autonomous proliferation [33]–[35]. Therefore, we blocked apoptosis and JNK signaling in these mutant tissues and examined the contribution of these pathways to the neoplastic phenotype of imaginal discs predominantly mutant for *ESCRT-II* components.

**Figure 5:**
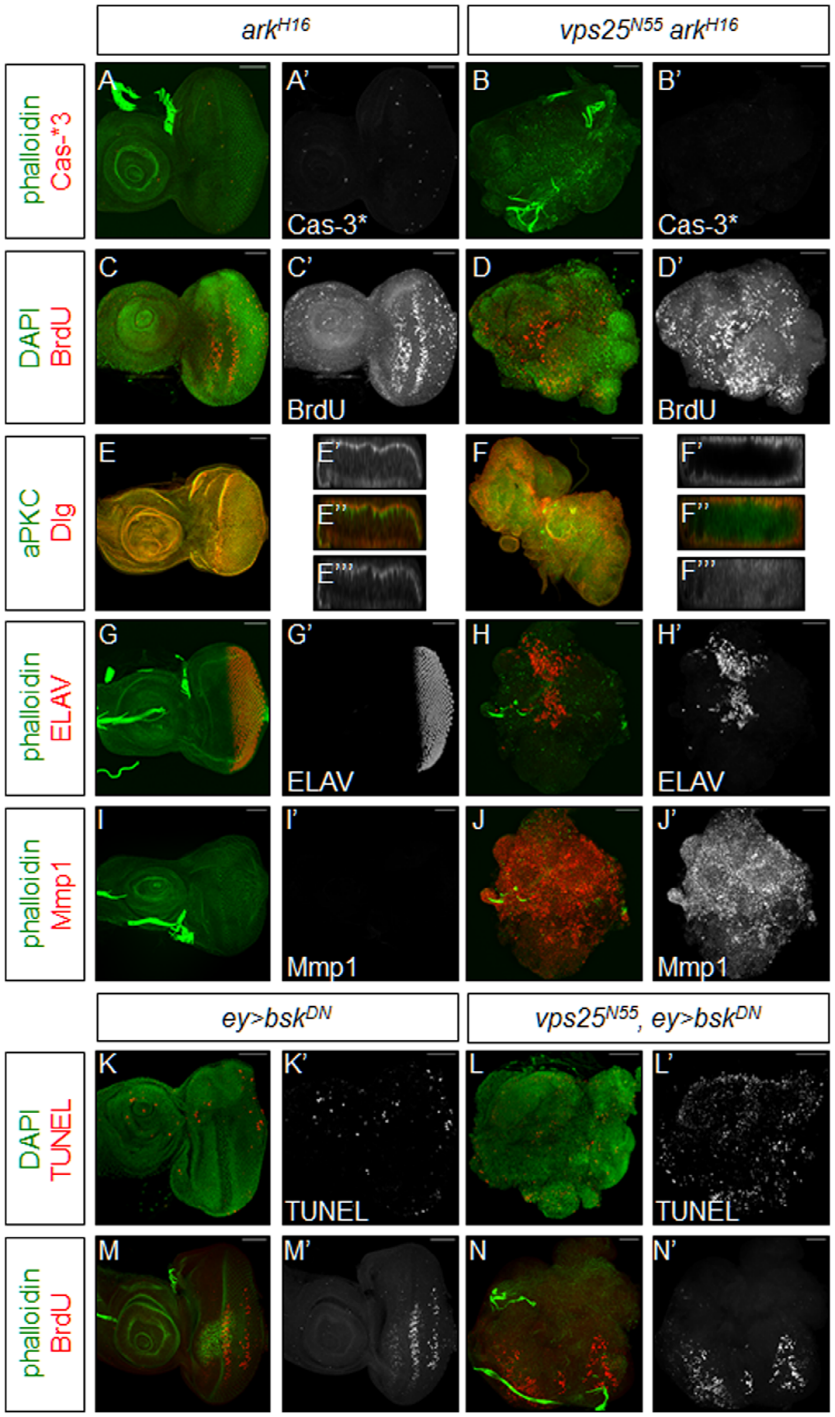
Inhibition of apoptosis affects the neoplastic transformation of ESCRT-II mutant tissue. Shown are predominantly mutant eye-antennal imaginal discs. Phalloidin (green) or DAPI (green) is used to mark the overall shape of the tissue. Scale bars represent 50 µm. (**A–J**) Apoptosis is inhibited by removal of *ark*. (**A,B**) Cleaved Caspase-3 (Cas-3*; red and grayscale) labelings show that apoptosis is completely inhibited in discs predominantly mutant for *vps25* and *ark* (B,B’). Cell death is also absent in discs predominantly mutant for *ark* (A,A’). (**C,D**) BrdU (red and grayscale) labelings show that proliferation is elevated in tissues predominantly mutant for *vps25* and *ark* (D,D’). Proliferation is not affected in control tissues predominantly mutant for *ark* (C,C’). (**E,F**) aPKC (red and grayscale (E’,F’)) and Dlg (green and grayscale (E’’’,F’’’)) labelings of discs predominantly mutant for *vps25* and *ark* show that cellular architecture is disrupted (F–F’’’). Cellular architecture is not disrupted in control discs predominantly mutant for *ark* (E–E’’’). (**G,H**) ELAV (red and grayscale) labelings of discs predominantly mutant for *vps25* and *ark* show that very few cells in the double mutant discs differentiate normally (H,H’). Differentiation occurs normally in control discs predominantly mutant for *ark* (G,G’). (**I,J**) Mmp1 (red and grayscale) labelings of discs predominantly mutant for *vps25* and *ark* show that levels of this protein are increased (J,J’). Mmp1 levels are not affected in control discs predominantly mutant for *ark* (I,I’). (**K–N**) Apoptosis is reduced by inhibition of JNK signaling by expression of the *UAS-bsk^DN^* transgene using *ey-Gal4*. Phalloidin (green) or DAPI (green) is used to mark the overall shape of the tissue. (K,L) TUNEL (red and grayscale) labelings show that apoptosis is reduced in discs predominantly mutant for *vps25* in which JNK signaling is inhibited (L,L’). Apoptosis is also low in control discs in which JNK signaling is inhibited (K,K’). (M,N) BrdU (red and grayscale) labelings show that proliferation is reduced in tissues predominantly mutant for *vps25* in which JNK signaling is inhibited (N,N’). Proliferation is not affected in control discs in which JNK signaling is inhibited (M,M’). **Genotypes**: (**A,C,E,G,I**) *eyFLP; FRT42D ark^H16^/FRT42D cl.* (**B,D,F,H,J**) *eyFLP; FRT42D vps25^N55^ ark^H16^/FRT42D cl.* (**K,M**) *eyFLP/UAS-bsk^DN^; FRT42D y^+^/FRT42D cl; ey-Gal4/+.* (**L,N**) *eyFLP/UAS-bsk^DN^; FRT42D vps25^N55^ y^+^/FRT42D cl; ey-Gal4/+*.

We first blocked apoptosis in mutant discs by generating discs that are predominantly double mutant for *vps25* and *ark*, the Apaf-1 related killer in flies that is an essential component of the cell death pathway [21], [36]–[40]. In *vps25 ark* double mutant discs, cell death is completely inhibited, as shown by Cas-3* labeling (Figure 5B). In these double mutant discs, the neoplastic phenotype is even more severe. In some animals, the two eye-antennal imaginal discs fuse together into one large epithelial mass (Figure 5F); in a few instances, the two brain lobes and two discs fuse together into a large mass. These tissue fusions were not observed in *vps25* single mutant discs and may indicate even more invasive behavior of apoptosis-inhibited *vps25* mutant tissue. High levels of proliferation, as indicated by BrdU incorporation, are consistent throughout the entire predominantly mutant tissues (Figure 5D). Cellular architecture is completely disrupted, as shown by the drastic spreading of aPKC and Dlg localization (Figure 5F). A few cells differentiate normally and thus are positive for ELAV, but most cells fail to differentiate (Figure 5H). Finally, there are high levels of Mmp1 throughout the mutant tissue, indicating that the tissue has the potential to be invasive (Figure 5J). Importantly, eye-antennal imaginal discs predominantly mutant for *ark* alone do not show any neoplastic characteristics (Figure 5C,E,G,I). Therefore, it is clear that cell death is not required for neoplastic transformation in tissues predominantly mutant for *ESCRT-II* components. In contrast, since the phenotype of *vps25 ark* double mutant discs is more severe than that of *vps25* single mutant discs (Figure 1), apoptosis in these mutant discs serves as a tumor suppressor mechanism to eliminate the cancerous tissue.

We also examined the role of JNK signaling in apoptosis, proliferation and neoplastic characteristics in discs predominantly mutant for *ESCRT-II* genes by inhibiting JNK signaling through overexpression of a dominant negative form of the *Drosophila* JNK homologue *basket* (*bsk*), *bsk^DN^* [27], using *ey-Gal4*. In control discs, overexpression of *bsk^DN^* in otherwise wild-type discs has no apparent effect on architecture, polarity, differentiation, and Mmp1 expression (Figure 5K,M, Figure S3A,C,E). However, compared to the apoptosis observed in *vps25* mutant discs (Figure 3C,G), TUNEL-positive cell death is strongly suppressed by expression of *bsk^DN^* in discs predominantly mutant for *vps25* (Figure 5L) suggesting that JNK signaling contributes to the apoptotic phenotype of predominantly mutant *ESCRT-II* eye discs. Intriguingly, the proliferation pattern is also reduced in these discs, as assayed by BrdU labeling (Figure 5N), implying that JNK-induced proliferation at least partially contributes to the strong proliferation phenotype of *vps25* mutant discs. Labeling with phalloidin (Figure 5N, Figure S3D,F) and staining with antibodies recognizing aPKC and Dlg (Figure S3B) both indicate that cellular architecture remains disrupted even when JNK signaling is inhibited. Mutant discs have lost their characteristic shape and instead are simply dense “balls” of cells. aPKC and Dlg are both spread outside of their normal domains of localization. Only a few cells in the disc are positive for the differentiation marker ELAV, and they are spread throughout the disc (Figure S3D). Finally, despite a report that JNK can induce *Mmp1* expression [35], expression of *bsk^DN^* in discs predominantly mutant for *vps25* does not suppress the elevated levels of *Mmp1* expression (Figure S3F), suggesting that other mechanisms can also induce Mmp1. Thus, while inhibition of JNK signaling partially blocks apoptosis and proliferation, is has no effect on the other neoplastic characteristics seen in *ESCRT-II* mutant cells.

### Inhibition of JAK/STAT Signaling Significantly Rescues the Neoplastic Transformation of *ESCRT*-*II* Mutant Tissues

**Figure 6:**
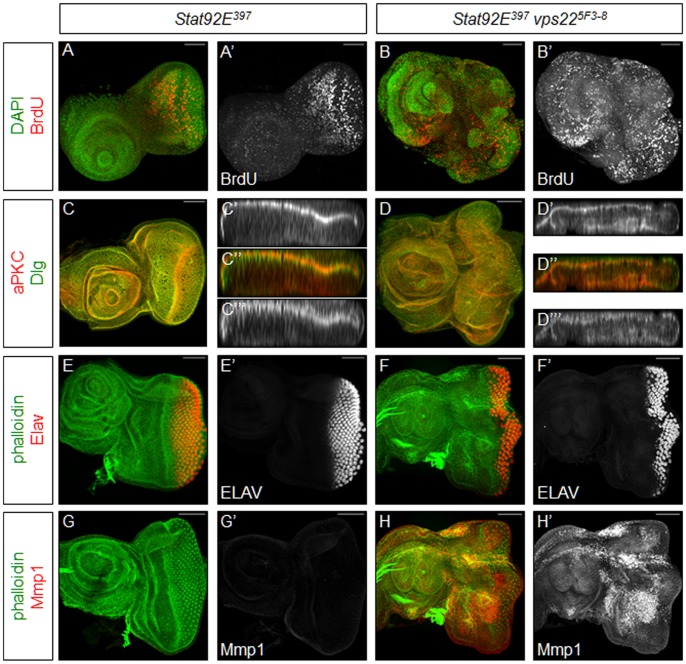
Inhibition of JAK/STAT signaling partially rescues the neoplastic transformation of ESCRT-II mutant tissue. Shown are predominantly mutant eye-antennal imaginal discs. Phalloidin (green) is used to mark the overall shape of the tissue. Scale bars represent 50 µm. (**A,B**) BrdU (red and grayscale) labelings show that proliferation is elevated in tissues predominantly mutant for *vps22* and *Stat92E* (B,B’) Proliferation is slightly abnormal in control tissues predominantly mutant for *Stat92E* (A,A’). (**C,D**) aPKC (red and grayscale (C’,D’)) and Dlg (green and grayscale (C’’’,D’’’)) labelings of discs predominantly mutant for *vps22* and *Stat92E* show that cellular architecture is largely intact (D–D’’’). Cellular architecture is not disrupted in control discs predominantly mutant for *Stat92E* (C–C’’’). (**E,F**) ELAV (red and grayscale) labelings of discs predominantly mutant for *vps22* and *Stat92E* show that differentiation is completely rescued (F,F’) from the loss of differentiation seen in ESCRT-II mutant discs. Differentiation occurs normally in control discs predominantly mutant for *Stat92E* (E,E’). (**G,H**) Mmp1 (red and grayscale) labelings of discs predominantly mutant for *vps22* and *Stat92E* show that levels of this protein are increased (H,H’). Mmp1 levels are not affected in control discs predominantly mutant for *Stat92E* (G,G’). **Genotypes**: (**A,C,E,G**) *eyFLP;; FRT82B Stat92E^397^/FRT82B cl.* (**B,D,F,H**) *eyFLP;; FRT82B vps22^5F3-8^ Stat92E^397^/FRT82B cl*.

Since we saw increased levels of JAK/STAT signaling in *ESCRT-II* mutant tissues, we investigated the possible autonomous role of JAK/STAT signaling in predominantly mutant tissues. A previous study examined *tsg101* mutant discs in a heterozygous *Stat92E* mutant background and reported a genetic interaction [18], but due to the heterozygous *Stat92E* condition, a rigorous analysis of the role of JAK/STAT signaling in the neoplastic transformation of nTSG mutant tissue has not been completed. To accomplish this, we completely inhibited JAK/STAT signaling in *vps22* mutant tissues using the null allele *Stat92E^397^*. We used *vps22* in these experiments because *vps22* and *Stat92E* both map to the same chromosome arm (3R), allowing a convenient double mutant analysis. It was recently shown that *Stat92E* mutant clones are eliminated by cell competition [41]. Interestingly, control discs predominantly mutant for *Stat92E* in which competitive interactions are eliminated reveal only weak abnormalities (Figure 6A,C,E,G). The proliferation pattern appears slightly abnormal (Figure 6A), and discs of slightly reduced size are generated. Importantly, overall tissue architecture (Figure 6A,E,G), apical-basal polarity (Figure 6C), and differentiation (Figure 6E) are normal in predominantly mutant *Stat92E* discs. There is also no Mmp1 expression in these discs (Figure 6G).

However, loss of JAK/STAT signaling in *vps22* mutant discs (*vps22 Stat92E* double mutants) strongly rescues the neoplastic characteristics seen in *vps22* single mutant tissues. The disorganization of cellular architecture observed in *vps22* mutant discs is significantly rescued by removal of JAK/STAT signaling. Labeling with phalloidin shows that double mutant discs retain their characteristic eye-antennal imaginal disc shape (Figure 6B,F,H). Staining with antibodies recognizing aPKC and Dlg reveals that spreading of these two proteins outside their wild-type domains of localization is minimized with most aPKC localized to the apical membrane domain and most Dlg localized to the basolateral membrane domain (Figure 6D). Thus, removal of JAK/STAT signaling leads to rescue of the disorganization of cellular architecture observed in *vps22* mutant tissues.

Loss of JAK/STAT signaling in discs predominantly mutant for *vps22* also significantly rescues the failure of differentiation seen in *vps22* mutant discs (Figure 6F). Few cells are positive for ELAV in *vps22* mutant discs, and cells that are differentiating normally are scattered throughout the tissue (Figure 1J–L). In striking contrast, when JAK/STAT signaling is inhibited, the entire posterior domain of the disc is positive for ELAV (Figure 6F), indicating that many cells are undergoing normal differentiation. This ELAV pattern is hardly distinguishable from the wild-type pattern (Figure 1I), implying that hyperactive JAK/STAT signaling in *vps22* mutant cells inhibits differentiation.

Loss of JAK/STAT signaling in *vps22* mutant discs, however, has little to no effect on Mmp1 expression. Mmp1 levels remain elevated throughout the tissue (Figure 6H), suggesting that JAK/STAT signaling is not required for Mmp1 expression and for possible metastatic capability. Thus, elevated JAK/STAT signaling in *ESCRT-II* mutant tissue plays a very important role in the neoplastic transformation, leading to both disorganization of cellular architecture and failure of differentiation.

## Discussion

While it is well established how de-regulated signaling pathways in *ESCRT*-*II* mutant clones mediate non-cell autonomous interactions with neighboring non-mutant cells to contribute to hyperplastic overgrowth and increased cell survival [1], [3], [5]–[9], [13], it was largely unknown which signaling pathways trigger neoplastic transformation autonomously. To address this question, we generated predominantly mutant eye-antennal imaginal discs in which competitive interactions are eliminated so that we could examine the autonomous results of de-regulated signaling.

Overall, it appears that the same signaling pathways that are induced in mosaic clones are also activated in predominantly mutant tissues. However, two results of this study are noteworthy. First, it is surprising that JNK activity is strongly induced in tissues predominantly mutant for *ESCRT-II* genes. This is surprising because JNK signaling was believed to be induced by cell competition from neighboring non-mutant cells in mosaic tissues [6], [16], [30]. However, non-mutant tissue is largely eliminated by the *ey-FLP/cl* method and thus competitive interactions are eliminated. Therefore, it is not known how JNK signaling is induced in these tissues. Nevertheless, JNK signaling is critical for the overgrowth phenotype of predominantly *ESCRT-II* mutant eye discs as inhibition of this pathway partially blocks cell proliferation. Second, de-regulation of the JAK/STAT signaling pathway is critical for the neoplastic transformation of *vps22* mutant discs. Loss of JAK/STAT signaling dramatically normalizes the neoplastic phenotype of *vps22* mutant cells. In addition to JNK and JAK/STAT activity, we also found Notch activity increased in discs predominantly mutant for *ESCRT-II* genes. Therefore, we tested a genetic requirement of Notch signaling for neoplastic transformation of *ESCRT-II* mutant cells. However, loss of *Notch* was inconclusive because even the wild-type control discs did not grow when Notch was inhibited (data not shown).

Interestingly, although *ESCRT-II* mutant tissues undergo neoplastic transformation, they also show high levels of apoptosis. Animals with predominantly mutant eye-antennal imaginal discs die as headless pharate pupae, a phenotype likely caused by the apoptosis of the imaginal discs before the adult stage. Reduction of JNK signaling in *vps22*, *vps25*, or *vps36* mutant discs leads to lower levels of apoptosis, supporting a role for JNK signaling in the cell death of the predominantly mutant tissues. More excitingly, JNK also controls proliferation in these tissues, as shown by the reduction of proliferation seen when JNK signaling was down-regulated. This observation is consistent with previous findings that JNK can induce non-cell autonomous proliferation [35] and that apoptosis-induced proliferation is mediated by JNK activity [33], [34], [42]. While inhibition of JNK signaling reduces proliferation in predominantly mutant *ESCRT-II* mutant discs, it does not affect other aspects of the neoplastic phenotype.

The role of JAK/STAT signaling in these mutants is complex. In mutant clones of *ESCRT-II* mosaic discs, Notch-induced secretion of the JAK/STAT ligand Upd triggers non-cell autonomous proliferation [5], [7]. However, we observed that autonomous de-regulated JAK/STAT signaling in predominately mutant discs is critical for the neoplastic transformation of *vps22* mutants. In *vps22 Stat92E* double mutant discs, organization of cellular architecture is definitively rescued with the layout of the tissue closely resembling that of a wild-type eye-antennal imaginal disc. In addition, apical-basal polarity markers are localized more-or-less correctly in these tissues, indicating that epithelial polarity is more intact. Finally, differentiation in the posterior portion of the eye disc is preserved when JAK/STAT signaling is inhibited. Thus, de-regulation of JAK/STAT signaling in *vps22* mutant discs contributes to the cellular disorganization and the lack of differentiation seen in the tissues, which is consistent with a previous study that implicated JAK/STAT signaling in cell cycle control, cell size, and epithelial organization in *tsg101* mutant tissues [18].

It was recently shown that cells with strong gain of JAK/STAT activity transform into supercompetitors and eliminate neighboring cells with normal JAK/STAT activity by cell competition [41]. However, in mosaic discs, a supercompetitive behavior of *ESCRT-II* mutant cells has not been observed. In fact, these mutant cells are eliminated by apoptosis. Only if apoptosis is blocked in these cells, is a strong overgrowth phenotype with neoplastic characteristics observed [5], [6]. Thus, apoptosis can serve as a tumor suppressor mechanism to remove cells with potentially malignant JAK/STAT activity.

How endosomal trafficking specifically regulates JAK/STAT signaling and, thus, how blocking trafficking leads to increases in signaling pathway activity are interesting questions to answer in the future. It is possible that, like endocytic regulation of the Notch receptor, the endosomal pathway tightly regulates Domeless (Dome), the JAK/STAT pathway receptor. It has been shown previously that Dome is trafficked through the endocytic machinery and that this trafficking of Dome can affect the downstream output of the JAK/STAT signaling pathway [43], [44]. It is also possible that Notch-induced Upd secretion causes autocrine JAK/STAT signaling in these mutants. However, technical problems (knocking down *Notch* function both in wild-type and mutant tissue causes general problems in tissue growth) prevented us from examining this possibility.

It will be important to examine how de-regulated JAK/STAT signaling in *ESCRT-II* mutants causes neoplastic transformation. JAK/STAT signaling is known to be an oncogenic pathway in *Drosophila* [45]–[49] and in humans [50] but its downstream targets that promote tumorigenesis are not yet clear. JAK/STAT signaling may be feeding into other pathways that promote tumorigenesis, such as *dpp* signaling [45], or may be targeting other proteins involved in transformation, such as Cyclin D [46], [51]–[54].

A number of studies have implicated genes that function in endocytosis and endosomal protein sorting as tumor suppressors in human cancers. Most well known is *Tsg101*, as early studies showed that downregulation of *Tsg101* promotes the growth of mouse 3T3 fibroblasts in soft agar [55]. When these cells were injected into nude mice, they formed metastatic tumors [55]. However, later studies have shown conflicting results [56]–[58], and it is still unclear if *Tsg101* functions as a tumor suppressor in metazoans. Importantly, a number of studies have shown changes in expression of ESCRT components in human cancer cells, including changes in expression of ESCRT-I components Tsg101 [59]–[62] and Vps37A [63], [64] and ESCRT-III components Chmp1A and CHMP3 [63], [65] . Since the primary proteins that function in endocytosis and endosomal trafficking are conserved from yeast to humans, it is likely that our findings in *Drosophila* may have important implications for human disease.

## Supporting Information

Figure S1 **Notch protein levels are upregulated in** ***vps22*** **,** ***vps25*** **, and** ***vps36*** **mutant tissues.** Shown are predominantly mutant eye-antennal imaginal discs. Scale bars represent 50 µm. Phalloidin (green) is used to mark the overall shape of the tissue. Notch protein levels (red, grayscale) are shown by staining with an antibody recognizing the intracellular domain of the protein (α-N^intra^). Notch protein levels are increased in imaginal discs predominantly mutant for *vps22* (B), *vps25* (C), or *vps36* (D), as compared to Notch protein levels in control discs (A,A’). **Genotypes**: (**A**) *eyFLP;; FRT82B/FRT82B cl.* (**B**) *eyFLP;; FRT82B vps22^5F3-8^/FRT82B cl.* (**C**) *eyFLP; FRT42D vps25^N55^ y^+^/FRT42D cl.* (**D**) *eyFLP;; vps36^Δ69^ FRT80B/cl FRT80B*.
(TIF)

Figure S2 **JNK signaling is upregulated in tissues predominantly mutant for** ***vps25.*** Shown are predominantly mutant eye-antennal imaginal discs. Phalloidin (green) is used to mark the overall shape of the tissue. *puc-lacZ* is detected by β-gal labeling (red or grayscale). (**A-B**) Imaginal discs predominantly mutant for *vps25* induce high levels of *puc-lacZ* (B), as compared to *puc-lacZ* expression in control discs (A,A’). Scale bars represent 50 µm. **Genotypes**: (**A**) *eyFLP; FRT42D/FRT42D cl; puc-LacZ/+.* (**B**) *eyFLP; FRT42D vps25^N55^ y^+^/FRT42D cl; puc-LacZ/+*.
(TIF)

Figure S3 **Inhibition of JNK signaling does not affect the disorganization of cellular architecture, the failure of differentiation, and the invasive potential of** ***ESCRT-II*** **mutant tissue.** Shown are predominantly mutant eye-antennal imaginal discs. JNK signaling is inhibited by expression of the *UAS-bsk^DN^* transgene using *ey-Gal4*. Phalloidin (green) is used to mark the overall shape of the tissue. Scale bars represent 50 µm. (A,B) aPKC (red and grayscale (A’,B’)) and Dlg (green and grayscale (A’’’,B’’’)) labelings of discs predominantly mutant for *vps25* in which JNK signaling is inhibited show that cellular architecture is disrupted (B-B’’’). Cellular architecture is not disrupted in control discs in which JNK signaling is inhibited (A-A’’’). (C,D) ELAV (red and grayscale) labelings of discs predominantly mutant for *vps25* in which JNK signaling is inhibited show that very few cells in the mutant discs differentiate normally (D,D’). Differentiation occurs normally in control discs in which JNK signaling is inhibited (C,C’). (E,F) Mmp1 (red and grayscale) labelings of discs predominantly mutant for *vps25* in which JNK signaling is inhibited show that levels of this protein are increased (F,F’). Mmp1 levels are not affected in control discs in which JNK signaling is inhibited (E,E’). Genotypes: (A,C,E) *eyFLP/UAS-bsk^DN^; FRT42D y^+^/FRT42D cl; ey-Gal4/+.* (B,D,F) *eyFLP/UAS-bsk^DN^; FRT42D vps25^N55^ y^+^/FRT42D cl; ey-Gal4/+*.
(TIF)
