# Supplementary figures and images for: De-Regulation of JNK and JAK/STAT Signaling in ESCRT-II Mutant Tissues Cooperatively Contributes to Neoplastic Tumorigenesis
Source: PLoS One. 2013 Feb 13;8(2):e56021. doi: 10.1371/journal.pone.0056021 (PMC3572140; doi:10.1371/journal.pone.0056021)

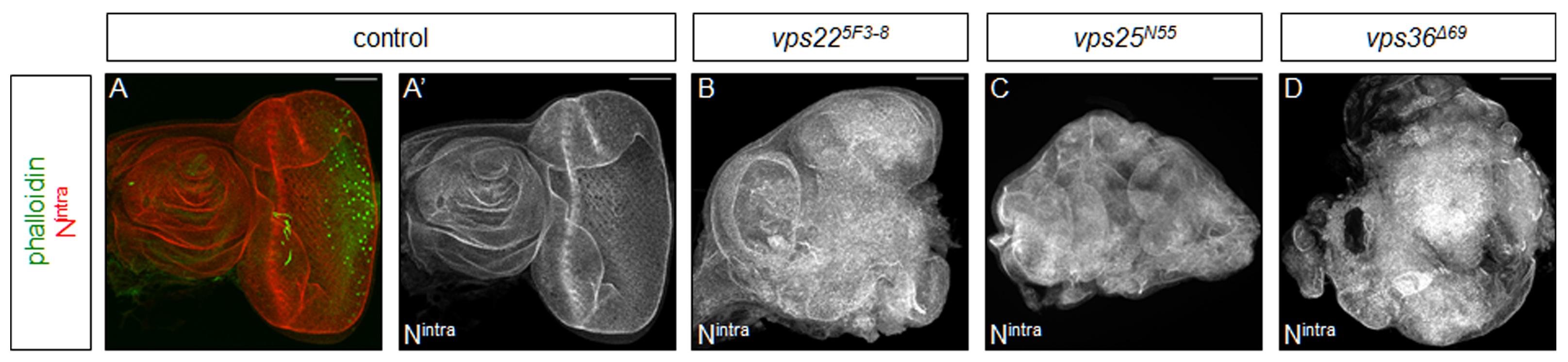

Supplement: Figure S1 — Notch protein levels are upregulated in vps22 , vps25 , and vps36 mutant tissues. Shown are predominantly mutant eye-antennal imaginal discs. Scale bars represent 50 µm. Phalloidin (green) is used to mark the overall shape of the tissue. Notch protein levels (red, grayscale) are shown by staining with an antibody recognizing the intracellular domain of the protein (α-Nintra). Notch protein levels are increased in imaginal discs predominantly mutant for vps22 (B), vps25 (C), or vps36 (D), as compared to Notch protein levels in control discs (A,A’). Genotypes: (A) eyFLP;; FRT82B/FRT82B cl. (B) eyFLP;; FRT82B vps225F3-8/FRT82B cl. (C) eyFLP; FRT42D vps25N55 y+/FRT42D cl. (D) eyFLP;; vps36Δ69 FRT80B/cl FRT80B. (TIF) [file pone.0056021.s001.tif]

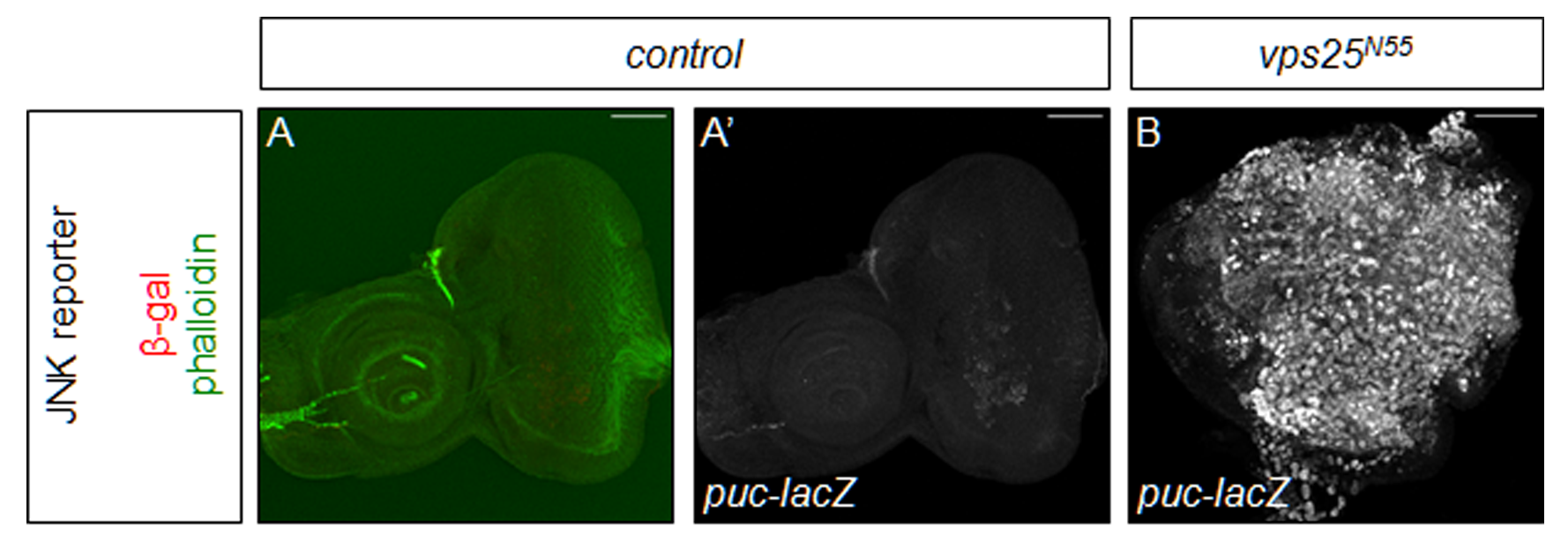

Supplement: Figure S2 — JNK signaling is upregulated in tissues predominantly mutant for vps25. Shown are predominantly mutant eye-antennal imaginal discs. Phalloidin (green) is used to mark the overall shape of the tissue. puc-lacZ is detected by β-gal labeling (red or grayscale). (A-B) Imaginal discs predominantly mutant for vps25 induce high levels of puc-lacZ (B), as compared to puc-lacZ expression in control discs (A,A’). Scale bars represent 50 µm. Genotypes: (A) eyFLP; FRT42D/FRT42D cl; puc-LacZ/+. (B) eyFLP; FRT42D vps25N55 y+/FRT42D cl; puc-LacZ/+. (TIF) [file pone.0056021.s002.tif]

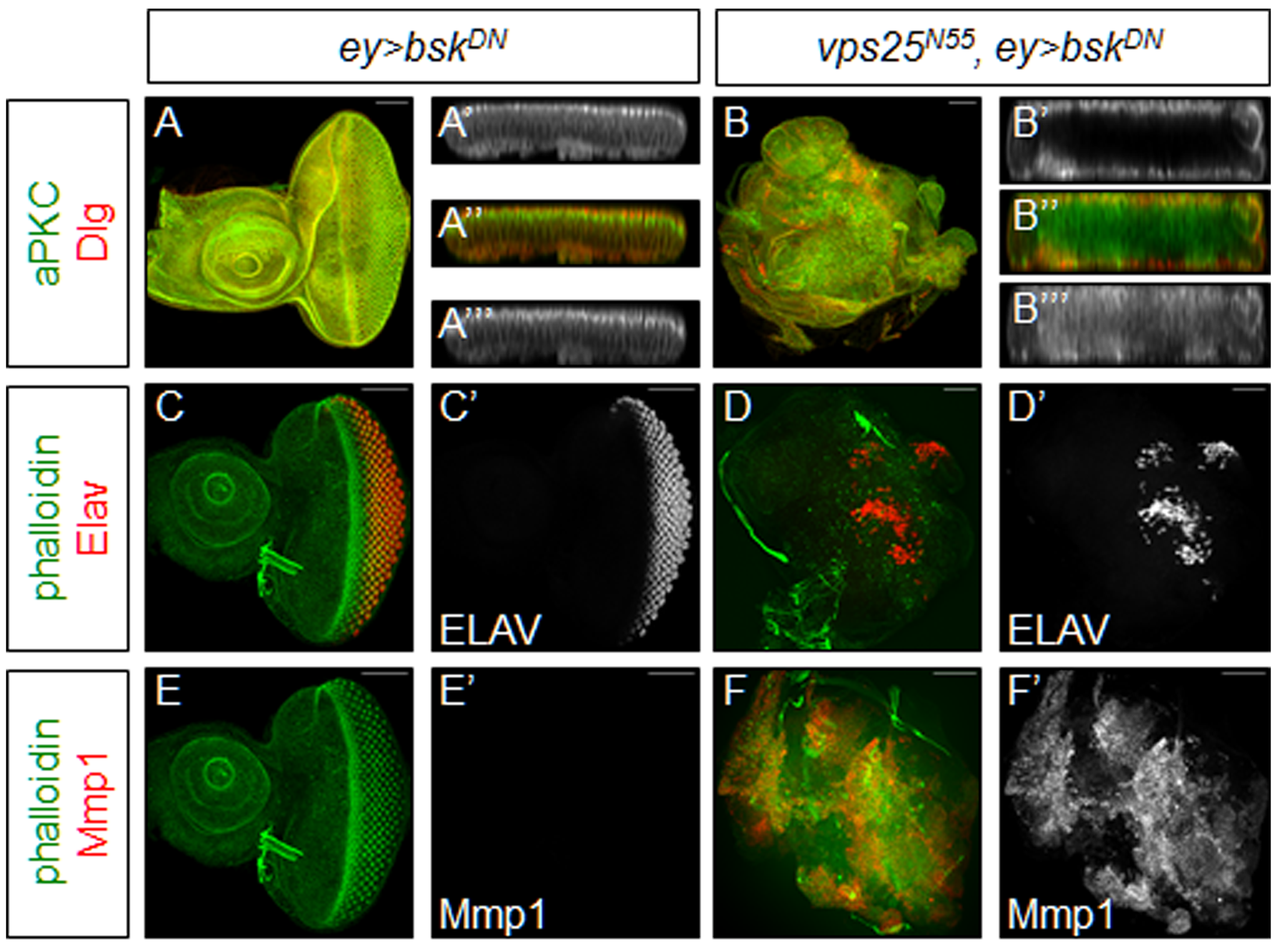

Supplement: Figure S3 — Inhibition of JNK signaling does not affect the disorganization of cellular architecture, the failure of differentiation, and the invasive potential of ESCRT-II mutant tissue. Shown are predominantly mutant eye-antennal imaginal discs. JNK signaling is inhibited by expression of the UAS-bskDN transgene using ey-Gal4. Phalloidin (green) is used to mark the overall shape of the tissue. Scale bars represent 50 µm. (A,B) aPKC (red and grayscale (A’,B’)) and Dlg (green and grayscale (A’’’,B’’’)) labelings of discs predominantly mutant for vps25 in which JNK signaling is inhibited show that cellular architecture is disrupted (B-B’’’). Cellular architecture is not disrupted in control discs in which JNK signaling is inhibited (A-A’’’). (C,D) ELAV (red and grayscale) labelings of discs predominantly mutant for vps25 in which JNK signaling is inhibited show that very few cells in the mutant discs differentiate normally (D,D’). Differentiation occurs normally in control discs in which JNK signaling is inhibited (C,C’). (E,F) Mmp1 (red and grayscale) labelings of discs predominantly mutant for vps25 in which JNK signaling is inhibited show that levels of this protein are increased (F,F’). Mmp1 levels are not affected in control discs in which JNK signaling is inhibited (E,E’). Genotypes: (A,C,E) eyFLP/UAS-bskDN; FRT42D y+/FRT42D cl; ey-Gal4/+. (B,D,F) eyFLP/UAS-bskDN; FRT42D vps25N55 y+/FRT42D cl; ey-Gal4/+. (TIF) [file pone.0056021.s003.tif]
